# Relation between psychosocial variables and the glycemic control of patients with type 2 diabetes: A cross-sectional and prospective study

**DOI:** 10.1186/1751-0759-3-4

**Published:** 2009-03-19

**Authors:** Takehiro Nozaki, Chihiro Morita, Sunao Matsubayashi, Koich Ishido, Hiroaki Yokoyama, Keisuke Kawai, Masahiro Matsumoto, Masato Takii, Chiharu Kubo

**Affiliations:** 1 Department of Psychosomatic Medicine, Graduate School of Medical Sciences, Kyushu University, 3-1-1 Maidashi, Higashi-ku, Fukuoka 812-8582, Japan; 2 Kitakyushu Municipal Medical Center, Kitakyushu, Japan; 3 Fukuoka Tokushukai Hospital, Fukuoka, Japan

## Abstract

**Background:**

This cross-sectional and prospective study used a variety of psychological inventories to evaluate the relationship between psychosocial factors and the glycemic control of patients with type 2 diabetes.

**Methods:**

Participants were 304 patients with type 2 diabetes who were treated as outpatients at diabetes clinics. All participants were assessed for HbA_1c _and completed the following self-report psychological inventories: 1) Diabetes Treatment Satisfaction Questionnaire (DTSQ), 2) Problem Areas in Diabetes Survey (PAID), 3) Well-being Questionnaire 12 (W-BQ12), 4) Self-Esteem Scale (SES), 5) Social Support Scale, and 6) Self-Efficacy Scale. HbA_1c _was again measured one year later. The relationships between the psychosocial variables obtained by analysis of the psychological inventories and baseline or one-year follow-up HbA_1c _were determined.

**Results:**

Baseline HbA_1c_was significantly correlated with age, diet treatment regimen, number of microvascular complication of diabetes, and the total scores of DTSQ, W-BQ12, PAID, SES and the Self-Efficacy Scale. Hierarchical stepwise multiple regression revealed that significant predictors of baseline HbA_1c _were total DTSQ and PAID scores, along with age, diet treatment regimen, and number of microvascular complication of diabetes after adjustment for demographic, clinical and other psychosocial variables. Two hundred and ninety patients (95.4% of 304) were followed and assessed one year after baseline. Hierarchical stepwise multiple regression analysis showed the significant predictors of follow-up HbA_1c _to be total DTSQ and PAID scores, along with age and diet treatment regimen. However, the correlation between baseline and follow-up HbA_1c _was so high that the only other variable to retain significance was diet treatment regimen once baseline HbA_1c _was included in the regression of follow-up HbA_1c_.

**Conclusion:**

The DTSQ and the PAID predicted both current and future HbA_1c _to a similar and significant degree in patients with type 2 diabetes.

## Background

The clinical course of diabetes, especially glycemic control, is largely influenced by patient self-management. The success of self-management is rather dependant on psychosocial aspects at the individual, interpersonal, social, and community levels. Fewer studies of the relationship between glycemic control and psychosocial factors have been conducted for type 2 diabetes than for type 1 diabetes. In cross-sectional research on type 2 diabetes, self-efficacy [1-3] and diabetes coping [4] were associated with good treatment adherence and good glycemic control, whereas stressful life events [5] and daily environmental stress factors [6] have been shown to be associated with poor metabolic control. Depression has been shown to have a significant association with increased HbA_1c _[7,8]. In other reports, anxiety was associated with hyperglycemia [9], diabetes-specific distress influenced poor adherence and poor glycemic control [10,11], and high self-esteem [12] and social support [13] were found to relate to good adherence. Moreover, the patient-physician interpersonal relationship was reported to be a contributor to better diabetes outcomes [14]. However, only a few longitudinal [1,15]studies have examined the relationship between psychosocial factors and glycemic control in type 2 diabetes.

The aim of the present study was to do a prospective and cross-sectional investigation to clarify the relationship between various psychosocial variables and glycemic control in Japanese patients with type 2 diabetes to determine the psychosocial variables most predictive of glycemic control.

## Methods

### Participants

Participants were 304 Japanese type 2 diabetes patients who were treated in outpatient diabetes clinics. Well-trained specialists in diabetes diagnosed patients according to the latest criteria of the Japan Diabetes Society: A fasting plasma glucose level of ≥ 126 mg/dl or an oral glucose-tolerance test with a 2-hour post-load plasma glucose level of ≥ 200 mg/dl. Causal plasma glucose > 200 mg/dl was also regarded as diabetes. The study was approved by the ethics committees of Kitakyushu City Hospital, Tokushukai Hospital, and Iki Municipal Hospital, all of which are central hospitals in the region. The aim of the present study, as described in the background section, was explained to the patients orally and in writing by their physicians, who also distributed and collected the questionnaire booklets and obtained written informed consent.

### Psychological measures

The questionnaire consisted of six psychosocial inventories.

1) The Diabetes Treatment Satisfaction Questionnaire (DTSQ) consists of eight items, each with seven possible answers. Six of the eight items are designed specially to measure a person's satisfaction with their diabetes treatment regimen [16]. Two additional items are concerned with the perceived frequency of hypo- and hyper-glycemia, which are scored separately as single items. In the present study, the total score of the six items was used as the treatment satisfaction scale. The range of total scores obtained by summing these six items from the DTSQ is from 0 to 36. We used the Japanese version of the DTSQ, which was linguistically and psychometrically evaluated by Ishii et al [17]. Cronbach's alpha for the total scores of the six items was 0.857 in the present study.

2) The Well-being Questionnaire 12 (W-BQ12) consists of 12 items and is a shortened version of the 22 item W-BQ that was designed to measure the psychological well-being of people with diabetes [18]. Items concerning somatic symptoms were avoided as they may lead to criterion contamination in populations with diabetes, where somatic symptoms such as fatigue or loss of appetite may be due to the physical condition of diabetes rather than to depression. We used the Japanese version of the W-BQ 12 that was linguistically and psychometrically evaluated by Riazi et al. [19]. Cronbach's alpha for the overall scale score in the present study was 0.928.

3) The Problem Areas in Diabetes Scale (PAID) is a measure of diabetes-specific emotional distress that was developed by Polonsky et al. [10] and translated into Japanese by Ishii et al. [20]. The scale consists of 20 items. There are five response options available for each PAID question: These responses are given a value from 0 (not a problem) to 4 (serious problem). According to the recommendation of the measure's authors, a total score was computed by summing the total item responses and multiplying this total by 1.25 to produce a total score that ranges from 0–100. Cronbach's alpha was 0.950 in the present study. 4) Self esteem was measured by use of the Rosenberg Self-esteem Scale (SES) [21]. The SES is a self-report questionnaire and consists of 10 items. Five of the items are phrased as positive and five as negative statements. Each item is answered according to a five-point scale, where a higher total score represents higher self-esteem. We used the Japanese version of the SES for which high internal consistency and construct validity have been confirmed [22]. Cronbach's alpha was 0.736 in the present study.

5) The Social Support Scale for patients with chronic disease (heart disease, diabetes mellitus, hypertension, etc.) was developed in Japan by Kim et al. [23]. The scale consists of 20 items related to social support in daily life for chronic disease and is divided into two subscales: emotional support in daily life and behavioral support for disease. Each item is scored on a Likert scale from 1 (not at all) to 4 (very applicable). The total score range extends from 4 to 80. A higher score represents higher social support in daily life. This test has high reliability and validity for each scale. Cronbach's alpha for the total score was 0.954 in the present study.

6) The Self-Efficacy Scale for health behaviors that influence health promotion in chronic disease patients was developed in Japan by Kim et al. [24]. The scale consists of 20 items and two subscales: active coping behavior with disease and controllability for health. Each item is scored on a Likert scale from 1 (not at all) to 4 (very applicable). The score range extends from 4 to 80. A higher score represents higher self-efficacy in dealing with health behavior. This test has high reliability and validity for each scale. Cronbach's alpha for the total score was 0.928 in the present study.

### Clinical variables

Demographic and clinical data were collected from medical records on the date closest to the start of the study. The HbA_1c _level was measured using high-performance liquid chromatography, with a normal reference range of 4.3%–5.8%. The diabetes treatment regimen consisted of either diet only or medication, which included oral hypoglycemic agents (OHA) or insulin or insulin plus OHA. The presence of microvascular complications of diabetes was determined as follows: 1) diabetic neuropathy was defined by clinical symptoms or by neurological examination; 2) diabetic retinopathy was diagnosed and assessed by an ophthalmologist; 3) diabetic nephropathy was diagnosed as the presence of prolonged microalbuminuria defined according to the consensus statement of the American Diabetes Association [25].

### One-year follow-up data collection

Of the 304 patients who completed the questionnaire at baseline, 32 were referred to local clinics near their residence because they had relatively good glycemic control (mean HbA_1c _= 6.8%). Six of these 32 patients dropped out of treatment at the referred clinic. Four patients in our central hospitals also dropped out of treatment and four patients died within one year. Thus, 290 patients followed for one year had complete data available for analysis (follow-up rate 96.5%). The demographic, clinical and psychosocial data at baseline were not significantly differentbetween the 290 patients who were followed for the whole year and the 14 patients who dropped out, whether calculated by the total of 14 or divided as 10 continuing patients and four deceased patients.

### Statistical analysis

Pearson's simple correlation test was done to search for associations between HbA_1c _and demographic, clinical, and psychosocial variables and for the correlation of each psychosocial variable. A hierarchical multiple regression analysis was done as a model for predicting current (baseline) and future (one-year follow-up) HbA_1c_. The following sequence was followed: first, demographic and clinical variables were entered as model 1; second, all psychosocial variables other than DTSQ or PAID score were added to model 1 as model 2 or model 3. Because DTSQ and PAID scores had a relatively high correlation, they were entered separately in the model to avoid multicollinearity; third, baseline HbA_1c _was added to models 2 and 3 as models 4 and 5 for predicting one-year follow-up HbA_1c_. Dummy codes were applied to sex, male = 1 and female = 0, and to treatment regimen, diet only = 1 and medication = 0, in Pearson's correlation test and multiple regression analysis. Comparison of demographic, clinical, and psychosocial variables between patients followed or not followed for one year was done by Student's t-test or χ^2 ^test. Comparison between baseline and follow-up HbA_1c _was done by Student's paired t-test. Cronbach's alpha was used to assess the internal consistency of the total score of each psychological test. All statistical analyses were performed with the SPSS software package (version 12.0J for Windows). Statistical significance was set at 0.05.

## Results

Table 1 shows the baseline demographic, clinical and psychosocial characteristics of our patients with type 2 diabetes. There were no significant differences in any demographic, clinical, or psychosocial factors between patients followed for one year (n = 290) and those who could not be followed (n = 14). Also, even after eliminating the four deceased patients, there were no significant differences in any of the demographic, clinical, or psychosocial variables between the remaining 10 patients who were not followed for the full year and the 290 patients who were. Moreover, although six of 10 patients who dropped out were referred to local clinics because of good glycemic control, there were no significant differences in any variables between the 290 patients completing follow-up and the six referred patients. In addition, of the 32 patients who were referred to local clinics, the 6 dropouts and 26 patients who did not drop out had no significant differences in any of the variables (data not shown).

**Table 1:** Baseline demographic, clinical and psychosocial characteristics of patients with type 2 diabetes

	Total	followed for one year (a)	not followed (n = 14)	
			
Baseline variables	(n = 304)	(n = 290)	dropouts (n = 10)	died (n = 4)
Demographic variables				
Sex(M/F)	170/134	161/129	6/4	3/1
Age (y)	61.9 ± 11	61.1 ± 11	57.4 ± 12	70.5 ± 8.2
Duration of illness (y)	11.6 ± 8.1	11.6 ± 10	8.8 ± 7.2	14.0 ± 3.6
Clinical variables				
HbA_1c _(%)	7.30 ± 1.2	7.32 ± 1.2	6.72 ± 0.88	7.83 ± 1.3
Treatment regimen (diet only/medication)	63/241	59/231	4/6	0/4
No. of microvascular complications of diabetes (0/1/2/3)	116/79/60/49	107/77/60/46	9/0/0/1	0/2/0/2
Psychosocial variables (score)				
DTSQ	25.3 ± 6.4	25.2 ± 6.4	28.0 ± 5.5	26.3 ± 7.4
W-BQ12	23.8 ± 6.7	23.8 ± 6.6	25.5 ± 8.1	20.0 ± 9.1
PAID	33.0 ± 21	33.4 ± 21	21.5 ± 23	37.2 ± 25
SES	35.2 ± 6.2	35.3 ± 6.2	32.6 ± 6.9	38.3 ± 5.4
Social Support Scale	56.2 ± 17	56.4 ± 16	48.1 ± 18	61.3 ± 22
Self-Efficacy Scale	74.0 ± 12	73.9 ± 12	75.9 ± 13	76.0 ± 8.8

Values are mean ± SD or n. Medication, oral hypoglycemic agents, or insulin, or insulin plus oral hypoglycemic agents. DTSQ, Diabetes Treatment Satisfaction Questionnaire measures satisfaction with the current therapy for diabetes; W-BQ12, Well-being Questionnaire12 measures the psychological well-being of patients with diabetes; PAID, Problem Areas in Diabetes Scale measures diabetes-specific emotional distress; SES, Self-Esteem Scale measures global self-esteem; Social Support Scale measures the degree of social support in daily life for chronic disease; Self-Efficacy Scale measures self-efficacy in dealing with health behavior in chronic disease.

Table 2 shows a correlation matrix of the total scores of the psychosocial inventories of the 304 patients at baseline. The DTSQ had significant, positive correlations with the W-BQ12 (correlation coefficient (r) = 0.254), SES (r = 0.128), Social Support Scale (r = 0.176), and Self-Efficacy Scale (r = 0.448) and a negative correlation with the PAID (r = -0.489). The W-BQ12 had significant positive correlations with the SES (r = 0.479), Social Support Scale (r = 0.314), and Self-Efficacy Scale (r = 0.525) and a significant negative correlation with PIAD (r = -0.555). The PAID score revealed significant negative correlations with all the other psychosocial variables, including the SES (r = -0.322), Social Support Scale (r = -0.169) and the Self-Efficacy Scale (r = -0.437). The SES showed a significant positive correlation with the Social Support Scale (r = 0.273) and the Self-Efficacy Scale (r = 0.401). A positive, significant correlation was found between the Social Support Scale and the Self-Efficacy Scale (r = 0.404).

**Table 2:** Correlation matrix of psychosocial variables at baseline (n = 304)

	Psychosocial variable					
	
Psychosocial variable	1	2	3	4	5	6
1 DTSQ	-					
2 W-BQ12	0.254**	-				
3 PAID	-0.489**	-0.555**	-			
4 SES	0.128**	0.479**	-0.322**	-		
5 Social Support Scale	0.176**	0.314**	-0.169**	0.273**	-	
6 Self-Efficacy Scale	0.448**	0.525**	-0.437**	0.401**	0.404**	-

Value is correlation coefficient (r). **P < 0.01.

DTSQ, Diabetes Treatment Satisfaction Questionnaire; W-BQ12, Well-being Questionnaire12;

PAID, Problem Areas in Diabetes Scale; SES, Self-Esteem Scale. Each measure is detailed in the footnotes of Table 1.

Table 3 displays the results of correlation coefficients by Pearson's simple correlation test and standard regression coefficients (β) by hierarchical stepwise multiple regression for the demographic, clinical and psychosocial variables and HbA_1c _at baseline. Baseline HbA_1c _was found to have significant positive correlations with, the number of diabetic complications (r = 0.168) and the baseline PAID score (r = 0.220), meaning that greater distress on diabetes was associated with a worse HbA_1c _value. In contrast, baseline HbA_1c _was found to have significant negative correlations with age (r = -0.311), diet treatment regimen (r = -0.249), and the baseline scores of the DTSQ (r = -0.219), W-BQ12 (r = -0.125), and Self-Efficacy Scales (r = -0.187), meaning that greater satisfaction, well-being and self-efficacy on diabetes were associated with a better HbA_1c _value. The hierarchical stepwise multiple regression model revealed that age, diet treatment regimen, and number of diabetes complications were significantly and independently associated with baseline HbA_1c _(Table 3, model 1, R^2 ^= 0.164). In model 2 (R^2 ^= 0.178), the DTSQ score was significantly associated with baseline HbA_1c _(β = -0.120, P = 0.03) after controlling for demographic, clinical, and psychosocial variables, except for PAID score. In model 3 (R^2 ^= 0.178), the PAID score was significantly related to baseline HbA_1c _(β = 0.131, P = 0.017) after controlling for demographic, clinical, and psychosocial variables, except for the DTSQ score. However, no more than 17.8% of the variance in current glycemic control was accounted for by age, diet treatment regimen, number of diabetes complications, and DTSQ or PAID score.

**Table 3:** Simple correlation coefficients (r) and standard regression coefficients by hierarchical stepwise multiple regression (β) between baseline variables and HbA_1c _at baseline of patients with type 2 diabetes (n = 304).

			Hierarchical stepwise regression analysis					
			
	Simple correlation		Model 1		Model 2		Model 3	
				
Baseline variables	r	P	β	P	β	P	β	P
			
Sex (male = 1, female = 0)	-0.047	0.148	-	-	-	-	-	-
Age	-0.311	<0.001	-0.316	<0.001	-0.296	<0.001	-0.295	<0.001
Duration of illness	0.051	0.376	-	-	-	-		-
Treatment regimen (diet only = 1, medication = 0)	-0.249	<0.001	-0.177	0.001	-0.155	0.005	-0.157	0.005
No. of microvascular complications of diabetes	0.168	<0.001	0.177	0.001	0.170	0.003	0.163	0.003
DTSQ	-0.219	<0.001	NI	NI	-0.120	0.030	NI	NI
W-BQ12	-0.125	0.030	NI	NI	-	-	-	-
PAID	0.220	<0.001	NI	NI	NI	NI	0.131	0.017
SES	-0.030	0.607	NI	NI	-	-	-	-
Social Support Scale	-0.052	0.367	NI	NI	-	-	-	-
Self-Efficacy Scale	-0.187	<0.001	NI	NI	-	-	-	-
Adjusted R^2^			0.164		0.178		0.178	
F			20.8	<0.001	17.2	<0.001	17.2	<0.001

Medication, oral hypoglycemic agents, or insulin, or insulin plus oral hypoglycemic agents. DTSQ, Diabetes Treatment Satisfaction Questionnaire; W-BQ12, Well-being Questionnaire12; PAID, Problem Areas in Diabetes Scale; SES, Self-Esteem Scale. Each measure is detailed in the footnotes of Table 1. R^2^, multiple coefficient of determination. NI, not included. Model 1, adjusted for sex, age, duration of illness, treatment regimen, number of microvascular complications of diabetes; Model 2, adjusted for Model 1 and DTSQ, W-BQ12, SES, Social Support Scale, Self-Efficacy Scale; Model 3, adjusted for Model 1 and W-BQ12, PAID, SES, Social Support Scale, Self-Efficacy Scale.

Table 4 displays the results by Pearson's simple correlation test and hierarchical stepwise multiple regression for baseline demographic, clinical and psychosocial variables and one-year follow-up HbA_1c_. HbA_1c _at the one-year follow-up was found to have significant positive correlations with the baseline PAID score and to have a significant negative correlation with age, diet regimen, and the baseline scores of the DTSQ, W-BQ12, SES and Self-Efficacy Scale. A highly significant positive correlation was found between baseline HbA_1c _and one-year follow-up HbA_1c _(r = 0.675, P < 0.01). In addition, HbA_1c _at the one-year follow-up (7.15 ± 1.2%) had significantly better values than the baseline HbA_1c _(7.32 ± 1.2%) of the 290 patients (P = 0.02 by paired t-test). The hierarchical stepwise multiple regression for one-year follow-up HbA_1c _as a dependent variable revealed that age and diet treatment regimen were significantly and independently associated with follow-up HbA_1c _(Table 4, model 1, R^2 ^= 0.127). In model 2 (R^2 ^= 0.138), DTSQ score was significantly associated with follow-up HbA_1c _(β = -0.115, P = 0.03) after controlling for demographic, clinical, and psychosocial variables, except for PAID score. In model 3 (R^2 ^= 0.144), PAID score was significantly related to follow-up HbA_1c _(β = 0.140, P = 0.014) after controlling for demographic, clinical, and psychosocial variables, except for DTSQ score. By adding baseline HbA_1c _to model 2, 47.9% of the variance in future glycemic control was accounted for by treatment regimen (β = -0.155, P = 0.001) and baseline HbA_1c _(β = 0.638, P < 0.001) in model 4. Also, by adding baseline HbA_1c _to model 3, 47.4% of the variance in future glycemic control was accounted for by treatment regimen (β = -0.155, P = 0.001) and baseline HbA_1c _(β = 0.634, P < 0.001) in model 5. The results of models 4 and 5 indicate that better current HbA_1c _and diet treatment are significant predictors of better future HbA_1c_. The statistically significant contribution of the DTSQ and the PAID to future HbA_1c _disappeared after adjustment for baseline HbA_1c_.

**Table 4:** Simple correlation coefficients (r) and standard regression coefficients by hierarchical stepwise multiple regression (β) between baseline variables and HbA_1c _at 1 year follow-up of patients with type 2 diabetes (n = 290).

			Hierarchical stepwise regression analysis									
			
	Simple correlation		Model 1		Model 2		Model 3		Model 4		Model 5	
						
Baseline variables	r	P	β	P	β	P	β	P	β	P	β	P
					
Sex (male = 1, female = 0)	-0.106	0.071	-	-	-	-	-	-	-	-	-	-
Age	-0.219	<0.001	-0.180	0.001	-0.153	<0.001	-0.154	0.001	-	-	-	-
Duration of illness	0.016	0.788	-	-	-	-		-	-	-	-	-
Treatment regimen (diet only = 1, medication = 0)	-0.318	<0.001	-0.294	<0.001	-0.278	0.001	-0.274	<0.001	-0.155	0.001	-0.155	0.001
No. of microvascular complications of diabetes	0.102	0.082	-	-	-	-	-	-	-	-	-	-
DTSQ	-0.202	0.001	NI	NI	-0.115	0.030	NI	NI	-	-	NI	NI
W-BQ12	-0.137	0.020	NI	NI	-	-	-	-	-	-	-	-
PAID	0.220	<0.001	NI	NI	NI	NI	0.140	0.014	NI	NI	-	-
SES	-0.095	0.108	NI	NI	-	-	-	-	-	-	-	-
Social Support Scale	-0.054	0.358	NI	NI	-	-	-	-	-	-	-	-
Self-Efficacy Scale	-0.166	0.001	NI	NI	-	-	-	-	-	-	-	-
HbA_1c_	0.675	<0.001	NI	NI	NI	NI	NI	NI	0.638	<0.001	0.634	<0.001
Adjusted R^2^			0.127		0.138		0.144		0.479		0.474	
F			22	<0.001	16.2	<0.001	17.0	<0.001	132	<0.001	130	<0.001

Medication: oral hypoglycemic agents, or insulin, or insulin plus oral hypoglycemic agents. DTSQ, Diabetes Treatment Satisfaction Questionnaire; W-BQ12, Well-being Questionnaire12; PAID, Problem Areas in Diabetes Scale; SES, Self-Esteem Scale. Each measure is detailed in the footnotes of Table 1. R^2^, multiple coefficient of determination. NI, not included. Model 1, adjusted for sex, age, duration of illness, treatment regimen, number of microvascular complications of diabetes; Model 2, adjusted for Model 1 and DTSQ, W-BQ12, SES, Social Support Scale, Self-Efficacy Scale; Model 3, adjusted for Model 1 and W-BQ12, PAID, SES, Social Support Scale, Self-Efficacy Scale; Model 4, adjusted for Model 2 and HbA_1c_at baseline; Model 5, adjusted for Model 3 and HbA1c at baseline.

Concerning treatment regimen, medication details (n = 231) are as follows: 74% of the patients on medication were treated with OHA (n = 171) and 26% were treated with insulin alone or insulin plus OHA (n = 60). After the one-year follow-up, the change of HbA_1c _in patients who were treated with diet only was from 6.71 ± 1.1% to 6.42 ± 0.82% (P = 0.022), the change in patients who were treated with OHA was from 7.32 ± 1.0% to 7.23 ± 1.1% (P = 0.265), and the change in patients who were treated with insulin or insulin plus OHA was from 7.93 ± 1.4% to 7.60 ± 1.4% (P = 0.012).

## Discussion

In the present study, the relation between psychosocial variables and glycemic control was investigated cross-sectionally and prospectively. The DTSQ and the PAID predicted both current and future HbA_1c _to a similar and significant degree in patients with type 2 diabetes.,, It has been reported that the PAID score of female patients with type 1 and type 2 diabetes who inject insulin is significantly related to HbA_1c _at one-year follow-up, even after adjustment for baseline HbA_1c _[11]. However, the amount of variance accounted for by the PAID in predicting one-year follow-up HbA_1c _was very small, whereas baseline HbA_1c _accounted for the majority of variance for follow-up HbA_1c _in the study. Therefore, the contribution of the PAID as predictor of future glycemic control does not seem so important. In the same way, the higher DTSQ score of the tablet-treated patients was significantly associated with current lower HbA_1c _[26]. No previous study has investigated whether or not the DTSQ is predictive of future glycemic control.

Both the DTSQ and the PAID, however, predicted future glycemic control in the present study when baseline HbA_1c _was not included in the regression of follow-up HbA_1c_. Baseline glycemic control was fair and the glycemic control was improved significantly after one year, but the degree of glycemic improvement was 0.17% of HbA_1c_, which was not so large. Under the usual treatment, the effect of psychosocial variables on future glycemic control could not help but being small because the correlation between current and future HbA_1c _is so high. Thus, in some situations where glycemic control is expected to improve much through behavioral or psychological therapeutic intervention or education programs, or alteration of treatment regimen [11,26,27], both DTSQ and PAID scales would predict the future glycemic condition regardless of current metabolic variables.

The only variable to retain significance in the regression of the follow-up HbA_1c _model was treatment regimen, even after adjustment for baseline HbA_1c_. This suggests that the patients with diet alone were appropriately treated, whereas the medication, including OHA, was not sufficient. In fact, patients who were treated with OHA did not improve over the year of the study, indicating that treatment of patients with OHA was insufficient.

It should be noted that the psychosocial variables dealt with in this study correlated significantly with each other. Especially, both the DTSQ and PAID found a relatively high inverse correlation. The focus of the PAID is on the extent to which people have negative experiences with no attention given to positive experiences, while the DTSQ deals with the full range of experiences of treatment, from very satisfied to very dissatisfied. We are better able to understand the psychology of patients with diabetes by measuring both positive and negative psychosocial aspects. Furthermore, because the DTSQ and PAID are associated with current and future HbA_1c _at almost the same level, both tests are very useful when we want to know the contribution of psychosocial factors to current and future glycemic control.

Nakahara et al. [15] recently reported a longitudinal assessment of the causal relationship between psychosocial variables and glycemic control in patients with type 2 diabetes using a structural-equation model. They found that self-efficacy directly reinforced adherence and that adherence had a direct positive relation to good future glycemic control. Diabetes-specific distress affected glycemic control indirectly through self-efficacy. This is reasonable because we would expect that those people who felt able to manage their diabetes well would be more likely to do so than people who felt they were not able to do so. In our study, therefore, the outcome that more emotional variables, such as satisfaction for diabetes treatment and diabetes-specific distress, are related to future glycemic control is meaningful.

The present study has several limitations. First, it is unclear concerning causal relationships among the psychosocial variables tested and glycemic control because a causal model was not applied. Second, we did not examine self-care or adherence, which is thought to influence glycemic control directly. Therefore, the detailed mechanisms of the psychosocial variables tested on glycemic control remain unknown. Third, we could not follow 14 patients for the full year, including four patients who died. It was thought likely that these patients were more dissatisfied with their treatment or were more distressed about their diabetes than the patients who completed the follow-up. However, there were no significant differences in any of the demographic, clinical, or psychosocial variables at baseline between patients followed for one year (n = 290) and those who could not be followed (n = 14) or even when eliminating the four deceased patients. Therefore, negligible bias was noted when the analysis was done with either 14 or 10 patients as dropouts.

## Conclusion

The DTSQ and the PAID predicted both current and future HbA_1c _to a similar and significant degree in patients with type 2 diabetes. However, the correlation between current and future HbA_1c _was so high that the only other variable to retain significance was treatment regimen when baseline HbA_1c _was included in the regression of future HbA_1c_.

## Competing interests

The authors declare that they have no competing interests.

## Authors' contributions

TN conceptualized and designed the study, collected and analyzed the data, interpreted the results, and drafted the manuscript. CM, SM and KI collected the data. All authors read and approved the final manuscript.
